# The transcription factor Nrf2 links Th2-mediated experimental allergy to food preservatives

**DOI:** 10.3389/fimmu.2024.1476480

**Published:** 2025-03-06

**Authors:** Yining Jin, Allison P. Boss, Jenna K. Bursley, Caitlin Wilson, Venugopal Gangur, Cheryl E. Rockwell

**Affiliations:** ^1^ Department of Pharmacology and Toxicology, College of Human Medicine Michigan State University, East Lansing, MI, United States; ^2^ Department of Food Science and Human Nutrition, Michigan State University, East Lansing, MI, United States; ^3^ Food Allergy & Immunology Laboratory, Department of Food Science and Human Nutrition, Michigan State University, East Lansing, MI, United States

**Keywords:** food additive, tBHQ, food allergy, anaphylaxis, immediate hypersensitivity, IgE, cytokines

## Abstract

**Introduction:**

Immune-mediated adverse reactions to food allergens are rising at a striking rate, for reasons that are not completely understood. Our previous studies suggest that the stress-activated transcription factor Nrf2 (Nuclear factor erythroid 2 -related factor) promotes Th2 differentiation, while inhibiting Th1 differentiation.

**Methods:**

In the present studies, we investigated the effect of Nrf2 activation on sensitization and anaphylaxis in response to food allergen in BALB/c mice. Specifically, we determined the effect of the Nrf2 activator and common food preservative tBHQ (*tert*-butylhydroquinone) on immune response to food allergen in Balb/c mice and SCID mice that received either wild-type or Nrf2-deficient CD4 T cells.

**Results:**

Our results demonstrate that tBHQ strongly increases IgE sensitization to ovalbumin (OVA) with a concurrent increase in plasma IgG1 concentrations. In addition, tBHQ in diet also exacerbated anaphylaxis and increased mast cell degranulation. In a recall response, tBHQ promoted a type 2 T cell response. Notably, adoptive transfer studies in SCID recipient mice indicate that Nrf2 expression in CD4^+^ T cells is critical to sensitization and anaphylaxis in response to food allergen. Likewise, the effects of tBHQ on sensitization and challenge are dependent on Nrf2 expression in CD4^+^ T cells.

**Conclusion:**

Overall, these studies point to a key role for Nrf2 in the immune response to food allergen. In addition, this study shows that the common food preservative tBHQ promotes allergic sensitization and anaphylaxis in experimental food allergy.

## Highlights

- The synthetic food additive, tBHQ, promotes IgE sensitization in response to OVA.
- Anaphylaxis following oral challenge was exacerbated in mice on a tBHQ diet.
- The effects of tBHQ are dependent on expression of Nrf2 in CD4 T cells.

## Introduction

Food allergies are growing at alarming rate afflicting 8% of children and 10.8% of adults in the USA (1, 2). A similar increase has occurred in other industrialized countries as well (3–5). Allergic reactions trigger clinical symptoms ranging in severity that affect the gastrointestinal tract, the skin, and the airways as well as the severe life-threatening symptom of systemic anaphylaxis (1, 2, 6–9). Cow’s milk, chicken egg, peanut, soybeans, wheat, fish, shellfish, and tree nuts are responsible for most of the allergic reactions to foods in the United States (10).

The mechanisms underlying the reason why the prevalence of food allergy has been increasing are incompletely understood at present (11, 12). In addition to the genetic predisposition of an individual, factors such as hygiene and lack of exposure to microbes, the composition of the intestinal microbiota, diet, obesity, Vitamin D, and environmental chemical exposure, have all been proposed to contribute to the global rise of food allergies (3, 9, 13–15). Considerable studies are dedicated to understanding the pathophysiology of IgE-mediated food allergies, and more recently, research efforts have shifted from genetic-centric to synergistic reasoning incorporating environmental factors (13, 16, 17). A few studies have shown that food allergies are linked to the exposure of certain environmental factors such as pesticides, tobacco smoke, dietary antacid use, antibiotics, triclosan, and air pollution (18–20). However, our research suggests that food additives are one of the most important chemical exposures, with ingestion as the major pathway for human exposure, and have not been studied extensively thus far (21).

Nrf2 is a stress-activated transcription factor that we have previously shown to play a role in T cell activation and polarization (22–24). Specifically, we demonstrated that Nrf2 activated by tBHQ promotes murine CD4^+^ T-cell differentiation towards a Th2 lineage in primary mouse CD4^+^ T cells, a key step in the development of allergy (23). Here, we show Nrf2 plays a critical role in mediating the allergic response to food antigen. We demonstrate that tBHQ in diet greatly increases allergen sensitization and exacerbates anaphylaxis and this effect is dependent on expression of Nrf2 in CD4^+^ T cells.

## Materials and methods

### Chemicals and reagents

Biotin conjugated rat anti-mouse IgE paired antibodies, IgG1 and isotype standards were purchased from BD BioSciences (San Jose, CA, USA); Albumin from chicken egg white was obtained from Sigma (St Louis, MO, USA). All other reagents were obtained from Sigma (St. Louis, MO) unless otherwise noted.

### Animals

All animal protocols are in compliance with the Guide for the Care and Use of Animals and were approved by the Institutional Animal Care and Use Committee (IACUC) at Michigan State University. Female 4-week-old BALB/c mice (The Jackson Laboratory, Bar Harbor, ME) were given control diet (AIN-93G special ordered with tBHQ omitted) (Dyets, Inc., Bethlehem, PA) or diet with 0.0014% tBHQ, 0.0014% butylated hydroxytoluene (BHT) or 0.0014% 3-hyrodxytyrosol (3-HT; AIN-93G, Dyets, Inc., Bethlehem, PA; where 0.0014% is the concentration of tBHQ that is normally included in the diet to prevent rancidification) one week prior to sensitization. The mice were then maintained on the diet for the duration of the study.

Mice were sensitized to sterile saline or ovalbumin as described below. Female BALB/c 3-week-old SCID (CBySmn.CB17-Prkdc^scid^/J) immunodeficient mice were purchased from Jackson (The Jackson Laboratory, Bar Harbor, ME). Nrf2-null mice on a mixed C57Bl/6 and AKR background were generated by Dr. Jefferson Chan at the University of California San Francisco (10.1073/pnas.93.24.13943). The mice were backcrossed 8 generations on the Balb/c background and were found to be >98% congenic.

### Sensitization

**Figure 1:**
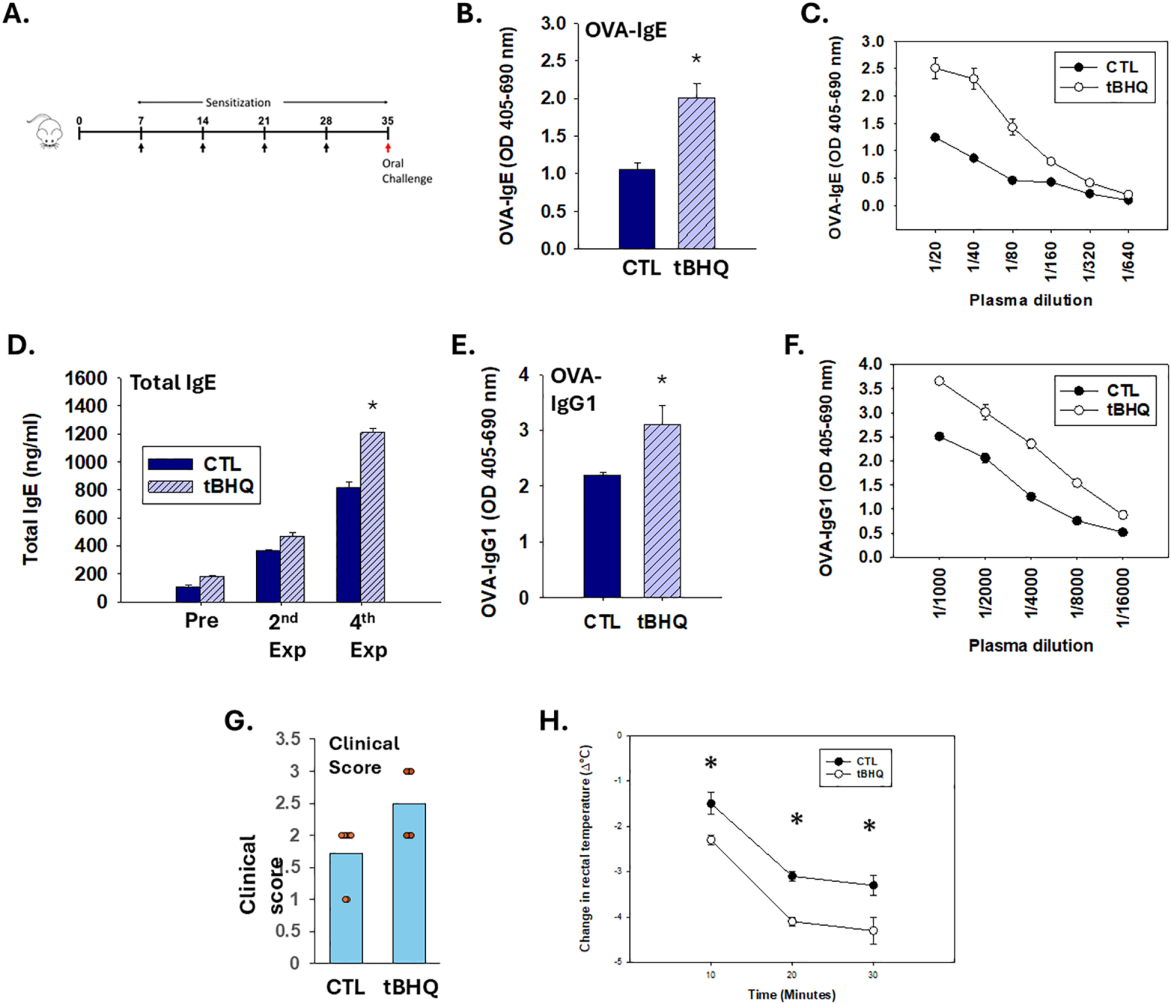
The Nrf2 activator, tBHQ, enhances sensitization and exacerbates anphylaxis to OVA. **(A-F)** Plasma antibody concentrations were quantified from mice on either a control diet (CTL) or tBHQ diet 4 weeks after OVA sensitization. **(A)** Schematic representation of the treatment protocol. **(B)** OVA-IgE levels calculated from individual plasma samples diluted 1/40; **(C)** OVA-IgE antibody titration from pooled plasma samples; **(D)** Total IgE plasma levels from individual animals; **(E)** OVA-IgG1 plasma levels quantified from individual animals at 1/2000 dilution; **(F)** OVA-IgG1 antibody titration from pooled samples. For pooled samples **(C, F)** data are presented as duplicate technical replicates. For individual samples **(B, D, E)** data are presented as mean ± SE. *Student’s t test: p < 0.05 as compared to CTL diet group (n=8). **(G, H)** Following sensitization, mice on either a control (CTL) or tBHQ diet were orally challenged with OVA (10 mg/mouse). Clinical symptoms were assessed for 30 min following challenge. **(G)** Clinical symptom scores are shown as a scatter plot with each symbol representing 1 mouse. Data are presented as mean ± SE. *Wilcoxon rank-sum test: p < 0.05 as compared to CTL diet group (n=6-7). **(H)** Rectal temperatures measured before and at every 10 min after oral challenge. Error bars denote standard error. *p < 0.05 as determined by two-way ANOVA with Bonferroni’s multiple comparisons test for *post hoc* analysis (n=6-7).

Dorsal skin was exposed by shaving the backs of the mice (*n* = 8/group) prior to transdermal sensitization to OVA (10mg/mouse) or sterile saline once per wk for 4 wks. Blood was collected from the saphenous vein into a lithium heparin pre-coated Microvette CB 300 Capillary Blood Collection Tube (Sarstedt, Newton, NC) on wks 2 and 4 after the first sensitization and the day of oral challenge (**Figure 1A**). Plasma was prepared from whole blood by centrifugation for 10 min at 2,000 x g at 4°C (Thermo Fisher Science Inc., Waltham, MA).

### Measurement of IgE and IgG1

OVA-specific IgE (OVA-IgE) and IgG1 (OVA-IgG1) were measured using optimized ELISA-based methods described previously (25, 26). The OVA-IgE and OVA-IgG1 levels were measured by coating ELISA plates with OVA (5 mg/mL) followed by blocking, sample addition, and detection using a biotin-labeled anti-mouse IgE or IgG1 detection antibody (BD Biosciences) as described previously (25). Total IgE was quantified by a sandwich ELISA using paired antibodies (i.e., a capture anti-mouse IgE antibody and a biotin-labeled anti-mouse IgE detection antibody) and a mouse IgE standard (BD Biosciences). OVA-IgE level in the tBHQ dose response study was performed by using Mouse OVA specific IgE ELISA kit (Biolegend, San Diego, CA).

### Oral challenge

The OVA-sensitized mice and saline-sensitized mice were challenged by oral gavage with OVA (10 mg/mouse). Rectal temperature (RT) was measured before and every 10 min up to 30 min after challenge using a temperature probe (ThermoWorks Inc., American Fork, UT). Mice were assessed by a blinded observer for signs of systemic anaphylaxis for 30 min following oral challenge. Clinical signs were evaluated according to a scoring system (27). Clinical scoring (on a scale of zero to 5) was performed according to the method described here. Scores were: 0 = no symptoms; 1 = scratching and rubbing around the nose and head; 2 = puffiness around the eyes and mouth, diarrhea, pili erecti, reduced activity and/or decreased activity with increased respiratory rate; 3 = wheezing, labored respiration, cyanosis around the mouth and the tail; 4 = no activity after prodding, or tremor and convulsions; 5 = death.

### Quantitation of mucosal mast cell protease-1 levels in the blood plasma

Blood samples were collected 1 h after oral challenge. Plasma levels of mouse mucosal mast cell protease-1 (mMCP-1) were measured using Mouse mMCP-1 ELISA kit (eBioscience Inc., San Diego, CA), according to the manufacturer’s instructions.

### Intracellular labeling for flow cytometry

Spleen cells (2 × 10^6^ cells/ml) in DMEM (Genesee Scientific, El Cajon, CA) containing 10% fetal bovine serum (Gibco), 25 mM HEPES (Sigma), 10 mM nonessential amino acids (Sigma), 100 u/mL penicillin-streptomycin solution (Sigma), and 55µM 2-mercaptoethanol (Invitrogen) were first stimulated with anti-CD3 (1.5 µg/ml) (eBioscience Inc., San Diego, CA)/anti-CD28 (1.5 µg/ml) (Invitrogen) and crosslinker AffiniPure F(ab’)_2_ Fragment Goat Anti-Syrian Hamster IgG(H+L) (1.5 µg/ml) (Jackson ImmunoResearch, West Grove, PA, USA) for 72 hours in the presence of 2 mM monensin (Biolegend, San Diego, CA), and then stained with viability dye (eBioscience Inc., San Diego, CA) and antibodies to cell surface markers, CD3-AF488 (Biolegend, San Diego, CA), CD4^+^-PE/Cy7 (eBioscience Inc., San Diego, CA) and CD8a-PerCP/Cy5.5 (Biolegend, San Diego, CA). Cells were then permeabilized with Foxp3 Staining Buffer Set (eBioscience Inc., San Diego, CA) according to the manufacturer’s instructions and stained for 30 min at room temp with anti-GATA3-APC (Biolegend, San Diego, CA) and anti-IL-4-PE (Biolegend, San Diego, CA). Isotype controls (intracellular) and single-color controls (surface) were used to set gates. Fluorescence was detected by an Attune NxT flow cytometer (Thermo Fisher Science Inc., Waltham, MA).

### OVA recall response and Th2 cytokine analysis

Single-cell splenocyte suspensions were prepared in DMEM (Genesee Scientific, El Cajon, CA) containing 10% fetal bovine serum (Gibco), 25 mM HEPES (Sigma), 10 mM nonessential amino acids (Sigma), 100 u/mL penicillin-streptomycin solution (Sigma), and 55µM 2-mercaptoethanol (Invitrogen). The cells (5×10^6^ cells/ml) were cultured in a flat-bottom 96-well plate (200 ul/well) (Greiner Bio-One, Monroe, NC) in the presence or absence of OVA (1mg/well) for 96 h (37°C, 5% CO_2_). IL-4, IL-5, and IL-13 were determined by the ELISA (eBioscience Inc., San Diego, CA) according to manufacturer’s instructions.

### Histopathology and mast cell degranulation analysis

Formalin-fixed duodenum tissues were processed by the Michigan State University Histopathology and Cytology Service Laboratory using standard methods described previously (28). Paraffin sections (Reichert Jung 2030 rotary microtome) were stained with toluidine blue. The numbers of mast cells (intact and degranulated) in 40 high-power fields were counted, and the percent degranulation was calculated as described earlier (28).

### Adoptive transfer experiments

CD4^+^ T cells were magnetically isolated from 6-week-old female wild-type or Nrf2-null mice on a BALB/c background (Milentyi Biotec). Likewise, B cells were isolated from 6-week-old female wild-type BALB/c mice. The cell isolations are performed in our laboratory routinely and typically result in >90% purity. 3-week-old female BALB/c SCID (CBySmn.CB17-Prkdc^scid^/J) immunodeficient mice (The Jackson Laboratory, Bar Harbor, ME) were recipients. 0.2 ml (5×10^6^ cells/ml) CD4^+^ T cells from the donor mice (wild-type or Nrf2^-/-^) were injected i.p. into the recipients. On the next day, 0.2 ml (5×10^6^ cells/ml) wild-type B cells were injected i.p. into the recipient mice. 8 wk after engraftment, mice were then sensitized for 6 wk and then challenged (29).

### PCR array

3-week-old female BALB/c SCID mice received B cells from wild-type BALB/c mice and CD4^+^ T cells from either wild-type (n=3) or Nrf2-null mice (n=3) on a BALB/c background following the protocol described in the adoptive transfer experiments and recipients were given on the control diet. 8 wk after engraftment, mice were sensitized for 6 wk. Mice were then sacrificed and total RNA was isolated from 4×10^6^ splenocytes using TRIzol Reagent per the manufacturer’s protocol (Thermo Fisher Science Inc., Waltham, MA). After isolation, reverse transcription was performed by using RT² First Strand kit (QIAGEN, Hilden, Germany). According to the manufacturer’s protocol, the cDNA was used on the real-time RT² Profile PCR Array Mouse Allergy & Asthma (QIAGEN, Hilden, Germany) in combination with RT^2^ SYBR Green ROX qPCR Mastermix (QIAGEN, Hilden, Germany). Relative mRNA expression was calculated by ΔΔCt, normalized to housekeeping genes (Actb, B2m, Gadph, Gusb, and Hsp90ab1) using Ct values quantified by ABI Quant Studio 6 Flex (Thermo Fisher Science Inc., Waltham, MA).

### DEGs and KEGG analysis

Differentially expressed genes (DEGs) between wild-type vs Nrf2-null group were identified as fold change >2 and P-values<0.05. Kyoto Encyclopedia of Gene and Genome (KEGG) analyses were performed based on the ClueGO, Cytoscape software. Pathway groups were generated based on Kappa Score = 0.5 and pathways with pV ≤ 0.05 were shown.

### Statistical analysis

For comparison of two groups (control diet and tBHQ diet), Student’s t-test was used (SigmaPlot 14 software). For studies with two factors (wild-type vs. Nrf2-null; control vs tBHQ diet), two-way ANOVA followed by Bonferroni’s or Holm-Sidak *post hoc* analysis was used (SigmaPlot 14 software). For comparison of multiple groups (tBHQ dose response), one-way ANOVA followed by Holm-Sidak *post hoc* analysis was used (SigmaPlot 14 software). Pearson correlation analysis was used to test the relationship between mMCP-1 levels and the drop in body temperature. An online software service was used in Pearson correlation analysis (http://www.socscistatistics.com/tests/). For comparison of clinical reactivity, statistical significance between two groups was analyzed by using the Wilcoxon rank-sum test and statistical analysis between multiple groups was performed using a Kruskal-Wallis test with Dunn’s *post hoc* test (SigmaPlot 14 software). Data are shown as mean ± SEM. For all analyses, statistical significance level was set at p < 0.05. DEGs and KEGG analysis is described separately (above).

## Results

### The food additive tBHQ promotes production of total and OVA-specific IgE and IgG1

To determine the effect of the food additive tBHQ, in the present studies, mice were transdermally exposed to OVA (10 mg/mouse) weekly for a total of 4 weeks (**Figure 1A**). Transdermal OVA sensitization induced a robust OVA-IgE antibody response by week 4 which was greater in the mice on the tBHQ diet (**Figures 1B–D**) (**Table 1**). Consistent with its effects on IgE, tBHQ also caused an increase in plasma OVA-IgG1 levels (**Figures 1E, F**) (**Table 1**).

**Table 1:** Determination of the titers of OVA-IgE and IgG1 antibodies in the plasma.

	OVA Specific Antibody Titer^a^	
Isotype	Control diet	tBHQ diet
OVA-IgE	320	640
OVA-IgG1	32,000	64,000

a The cut-off values used for each isotype were set as the mean value ± 3SD of negative controls (buffer only) (44).  Antibody titer is the reciprocal of the plasma dilution that shows positive binding to OVA in an ultra-sensitive ELISA based method (29); data shown is the geometric mean titer of duplicate analysis of pooled samples after 4 courses of sensitization (n=8 mice/group).

### Exacerbated clinical signs of anaphylaxis in mice on a tBHQ diet following OVA challenge

One week after the last sensitization, the mice were challenged orally to OVA (10 mg/mouse) and observed for clinical symptoms of anaphylaxis. Oral challenge of OVA-sensitized mice resulted in clinical signs of immediate hypersensitivity reaction, including inactivity, isolation, scratching/rubbing and increased respiratory rate with onset occurring almost immediately following challenge. Notably, mice on the tBHQ diet demonstrated more severe symptoms with an average clinical score around 2.5 (**Figure 1G**).

We also evaluated the hypothermia shock response (HSR) by measuring the drop in rectal temperature after oral allergen challenge. OVA-sensitized mice on both the control diet and the tBHQ diet showed a significant drop in rectal temperature with maximal drop at 30 min post oral challenge. The decrease in temperature was significantly more pronounced in mice on the tBHQ diet (2.3°C drop in tBHQ diet group vs. 1.5°C drop in control diet group at 10 min) (**Figure 1H**). There was no difference in baseline body temperatures between the groups (**Supplementary Figure 1**). Overall, these data indicate that the mice on a tBHQ diet show exacerbated clinical signs of food allergen-induced anaphylaxis as well as HSR upon oral allergen challenge.

### Increased numbers of mast cells in spleens of mice on the tBHQ diet

Because splenic immune responses play a role in the development of allergic diseases and spleen is a site where mast cells are induced in the development of food allergy, we next investigated mast cells in the spleen of allergic mice after oral challenge (30). The percentage of FcϵRI^+^/c-kit^+^ cells (mast cell population) was significantly higher in mice on the tBHQ diet compared to those on control diet, consistent with an increase in the number of mast cells (**Supplementary Figure 2**).

### tBHQ promotes robust mucosal mast cell degranulation in OVA-sensitized mice

**Figure 2:**
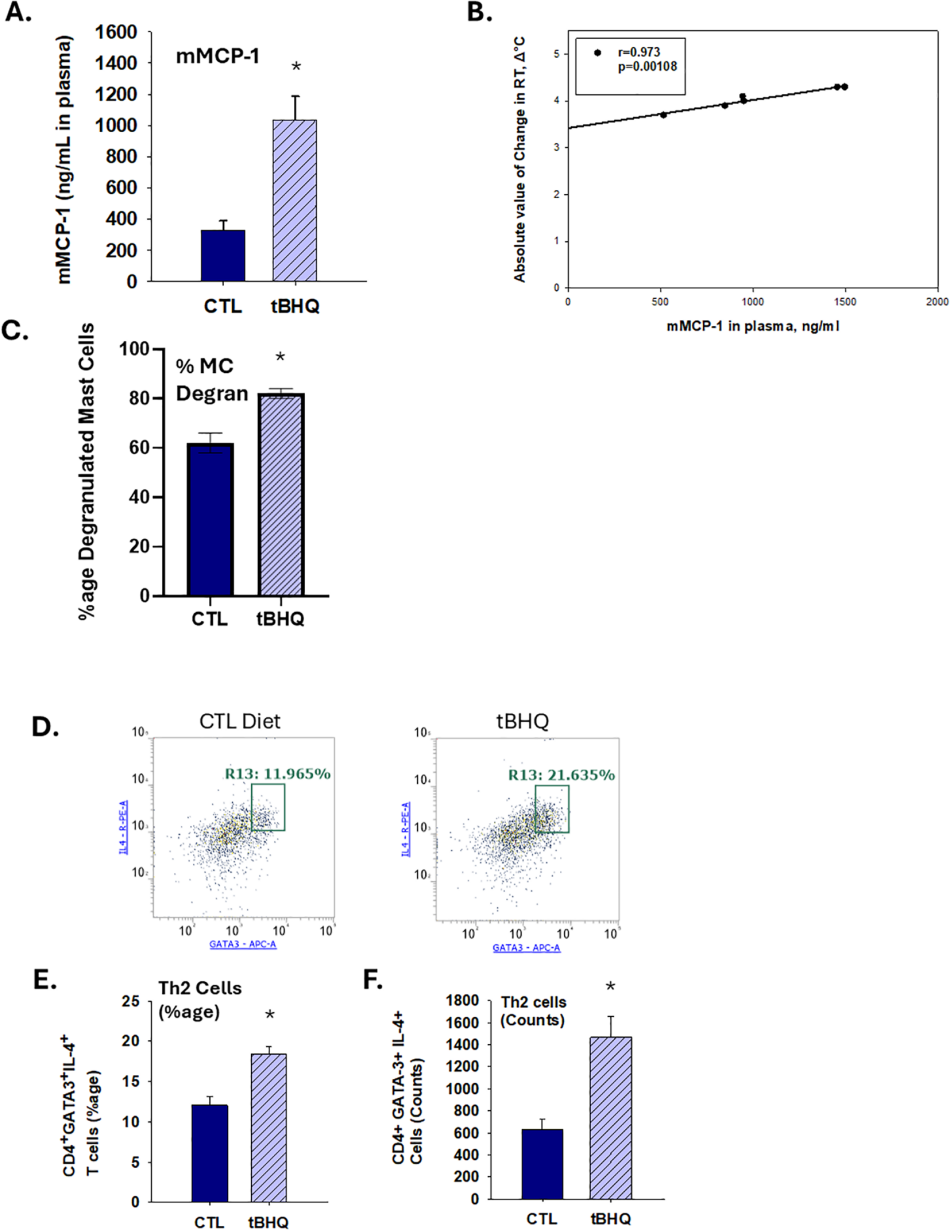
An increase in plasma levels of mast cell protease-1 and Th2 cell counts in tBHQ-fed mice occurs concurrently with worsened anaphylaxis. **(A)** Plasma levels of murine mast cell protease (mMCP)-1 1 h after oral challenge as determined by ELISA. **(B)** Pearson analysis of correlation between mMCP-1 plasma concentrations and body temperature 10 min post-challenge. Data are presented as mean ± SE. *Student’s t test: p < 0.05 as compared to CTL diet group at 1 h post-challenge. (n=6-7). **(C)** The percent degranulation of mast cells in duodenum as determined by toluidine blue staining. Toluidine blue positive metachromatic mast cells were counted in the tissue sections from OVA-sensitized mice on the tBHQ diet, control diet and saline-sensitized mice. In general, 2 sections per tissue per animal were examined under the microscope. Numbers of mast cells (intact and degranulated) in 40 high power fields (HPF) were counted and percent degranulation was calculated as the degranulation cell count over the total mast cell count. (n=3 mice/each group) *Student’s t test: p < 0.05 as compared to control diet (CTL, n=3). **(D)** Representative dot plots for Th2 cells (CD4^+^GATA-3^+^IL-4^+^) including percentages within gates for CTL diet versus tBHQ. **(E)** The percentage and **(F)** the absolute counts of Th2 cells among splenic CD4^+^ T cells from mice on either a CTL or tBHQ diet. Data are presented as mean ± SE. *Student’s t test: p < 0.05 as compared to control diet (CTL, n=4).

IgE-mediated mast cell degranulation is a critical event to initiate anaphylaxis. To quantify the extent of IgE-mediated mast cell degranulation, we measured plasma levels of mucosal mast cell protease-1 (mMCP-1). Oral challenge with OVA elicited a robust elevation of mMCP-1. Notably, mMCP-1 response to OVA challenge was 3-fold greater in mice on the tBHQ diet (**Figure 2A**). A Pearson analysis 0.973, p<0.05) suggests a correlation between mMCP-1 plasma levels and a drop in body temperature at 20 min post challenge (**Figure 2B**), indicating that mast cell degranulation is likely correlated to HSR. In addition, duodenum tissue sections were collected and stained with toluidine blue for detection of mast cells. Histopathology studies demonstrated increased numbers of degranulated mast cells in the duodenums of mice in the tBHQ group (**Figure 2C**, **Supplementary Figure 1**). Taken together, these data show that increased mast cell degranulation and elevated mMCP-1 levels in mice in the tBHQ group strongly correlates with significantly higher HSR and clinical symptom scores.

### tBHQ increases the number of IL-4 producing Th2 cells after oral challenge

Skewed Th2 polarization can promote IgE production and is associated with the development of food allergy. Moreover, our previously published studies demonstrate that tBHQ promotes Th2 polarization *in vitro* (23). Thus, we quantified the percentage of splenic Th2 cells in this study. Notably, following challenge, the percentage of splenic Th2 cells (CD4^+^ GATA-3^+^IL-4^+^ cells) was significantly increased in the tBHQ group (**Figures 2D–F**). Taken together, our previous and present studies combined suggest that tBHQ promotes Th2 polarization.

### Increased induction of Th2 cytokines by tBHQ in a recall response

**Figure 3:**
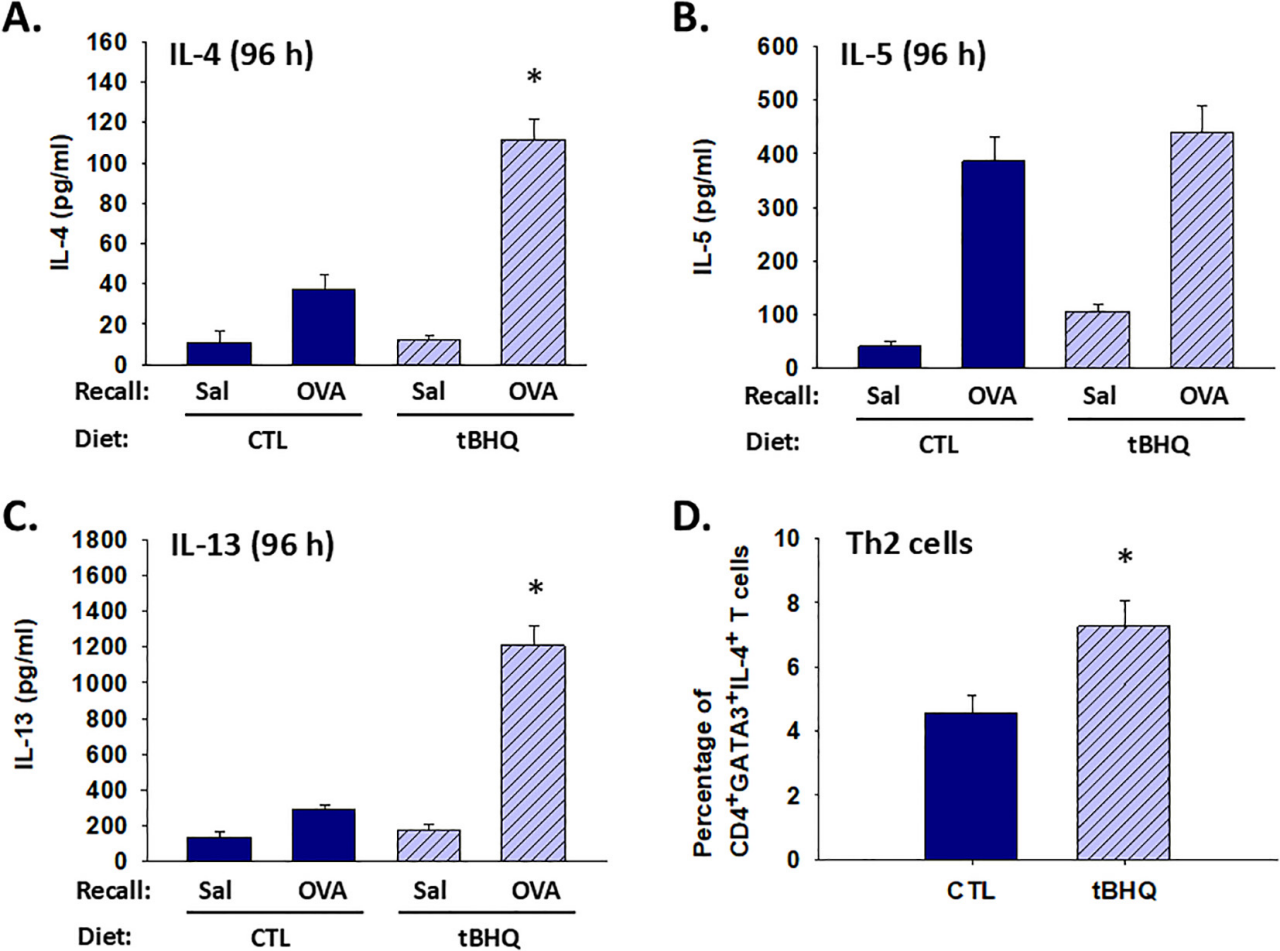
Mice on the tBHQ diet have a potentiated ex vivo recall response to OVA. Splenocytes were isolated from mice on either a control (CTL) or tBHQ diet following sensitization and oral challenge to OVA. The isolated splenocytes were cultured in the presence and absence of OVA (1mg/well) for 96 h in an ex vivo recall response. Quantification of **(A)** IL-4, **(B)** IL-5 or **(C)** IL-13 in cell supernatants by ELISA. **(D)** The percentage of CD4^+^GATA-3^+^IL-4^+^ cells (Th2 cells) among total splenocyte population. Data are presented as mean ± SE. *Student’s t test: p < 0.05 as compared to the CTL (n=4).

Th2 cytokines, such as IL-4, IL-5 and IL-13, have a number of effects associated with allergy, including IgE class-switching by B cells, eosinophil activation and recruitment, and mucus production (31). Thus, we quantified the ex vivo recall response to OVA in splenocytes derived from OVA-sensitized and challenged mice. We found that splenocytes from mice on the tBHQ diet produced 3-4 fold more IL-4 and IL-13 than splenocytes from mice on control diet (**Figures 3A, C**). Though IL-5 from OVA-restimulated splenocytes was not different between groups, there was a trend toward an increase in basal levels in the tBHQ group (**Figure 3B**). Likewise, we found an increased percentage of Th2 cells in the tBHQ group (7%) as compared to those from the control group (4.5%) (**Figure 3D**) but not in the saline control group (**Supplementary Figure 1**). These data further support the hypothesis that tBHQ promotes type 2 immune responses.

### The effect of tBHQ in the OVA-sensitized mice is dose-dependent

**Figure 4:**
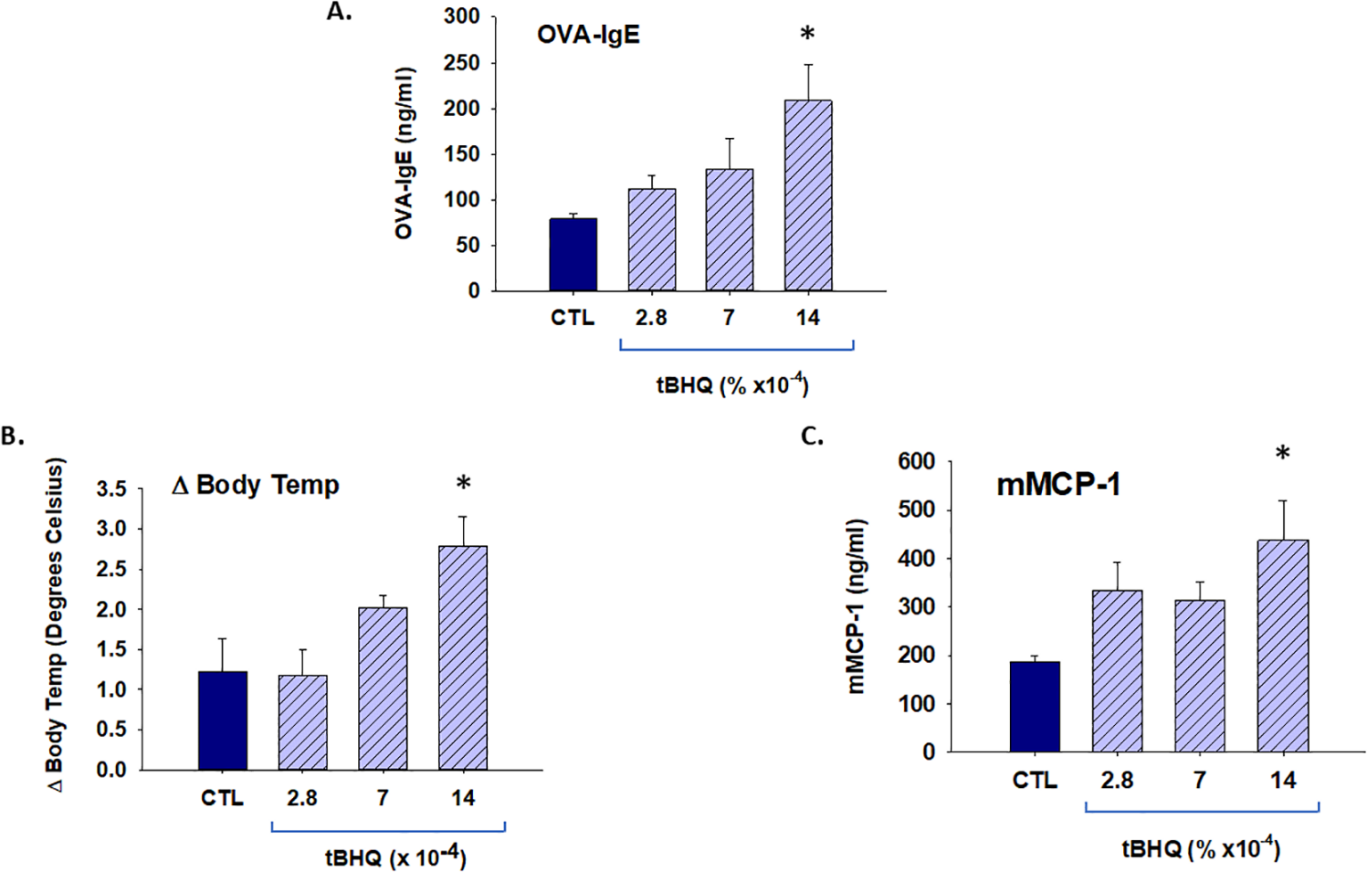
TBHQ increases allergic sensitization and anaphylaxis in a dose-responsive manner. Plasma antibody concentrations were quantified from mice on either a control diet (CTL) or tBHQ diet 4 weeks after OVA sensitization. **(A)** OVA-IgE levels calculated from individual plasma samples diluted 1/40. **(B)** Rectal temperatures 20 min after oral OVA challenge. **(C)** Plasma levels of murine mast cell protease (mMCP)-1 1 h after oral challenge as determined by ELISA. Error bars denote standard error. *p < 0.05 as determined by one-way ANOVA with Holm-Sidak test for *post hoc* analysis (n=5).

To determine the dose response of tBHQ on allergic sensitization and anaphylaxis, mice were fed AIN-93G with 0.0014% tBHQ (standard), 0.0007% tBHQ (50% of the standard), and 0.00028% tBHQ (20% of the standard) or control diet and exposed to OVA (10 mg/mouse) weekly for a total of 4 weeks. Sensitization with OVA elicited a more robust OVA-specific IgE antibody response in mice on the tBHQ diet at the highest concentration (0.0014%, the concentration found in standard chow, **Figure 4A**). One week after the last sensitization, the mice were challenged orally to OVA (10 mg/mouse) and body temperature and plasma levels of mMCP-1 were measured. Compared to the mice on the control diet, mice on the 0.0014% tBHQ diet exhibited a greater drop in body temperature and significantly elevated levels of mMCP-1, while 0.0007% tBHQ and 0.00028% tBHQ groups showed a non-significant trend toward an increase in mMCP-1 (**Figures 4B, C**). Overall, the data point to a dose-dependent effect of tBHQ on allergic sensitization and anaphylaxis.

### The effect of tBHQ on allergic sensitization and anaphylaxis is selective

**Figure 5:**
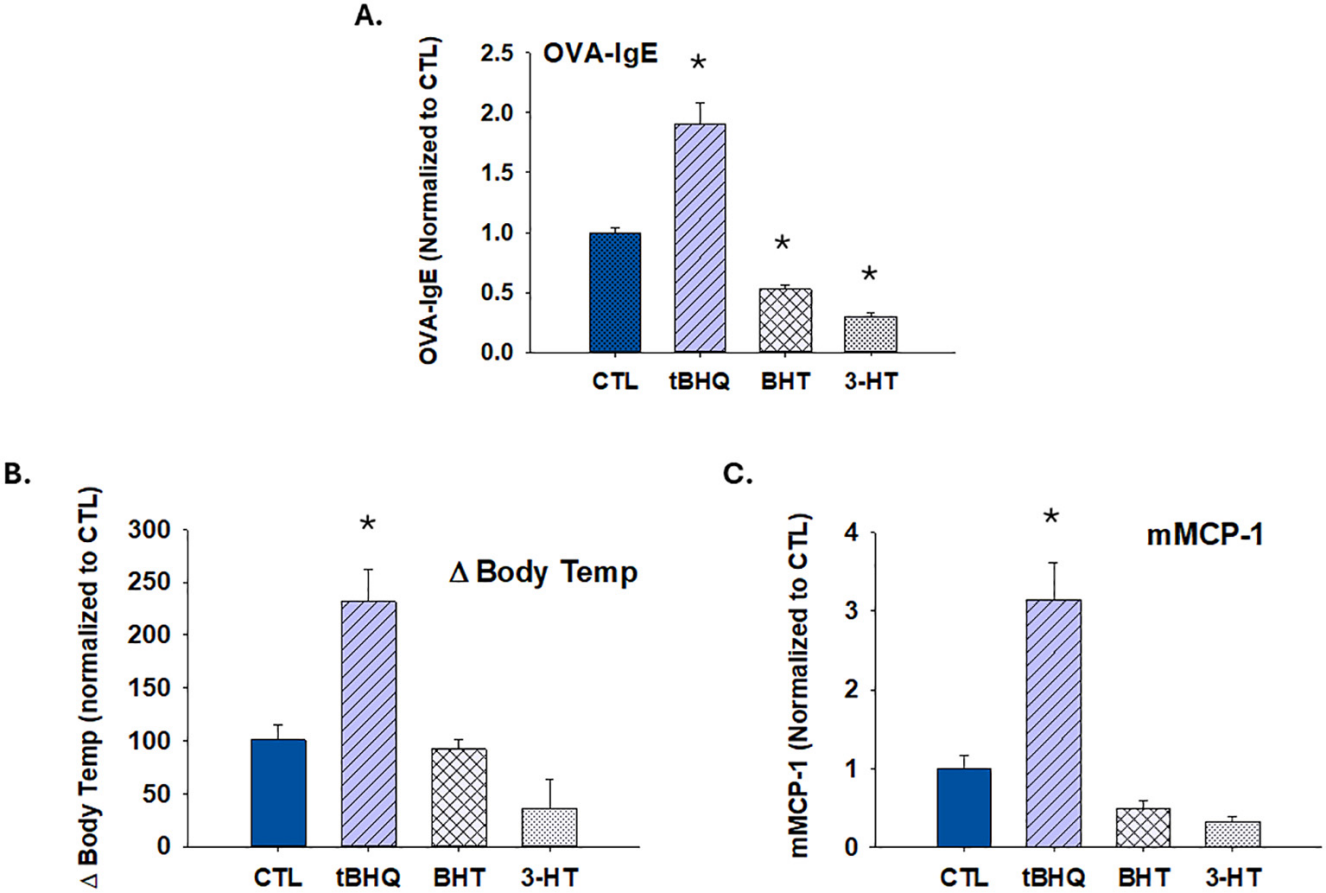
The increase in allergic sensitization and anaphylaxis by tBHQ is selective. Plasma antibody concentrations were quantified from mice on either a control (CTL), butylated hydroxytoluene (BHT), 3-hydroxytyrosol (3-HT) or tBHQ diet 4 weeks after OVA sensitization. **(A)** OVA-IgE levels calculated from individual plasma samples diluted 1/40. **(B)** Rectal temperatures 20 min after oral OVA challenge. **(C)** Plasma levels of murine mast cell protease (mMCP)-1 1 h after oral challenge as determined by ELISA. Error bars denote standard error. *p < 0.05 as determined by one-way ANOVA with Holm-Sidak test for *post hoc* analysis (n=5).

It is established that oxidative burden impacts immune cell polarization and many different disease states. To determine whether other antioxidants would have a similar effect to that of tBHQ, mice were exposed to additional antioxidants through diet. Butylated hydroxytoluene (BHT) is a synthetic antioxidant that is widely used as a food preservative. 3-hydroxytoluene (3-HT) is a naturally-occurring antioxidant found in olive oil. The effects of BHT and 3-HT were not similar to those of tBHQ. Whereas tBHQ enhanced IgE sensitization and exacerbated anaphylaxis, BHT and 3-HT did not (**Figure 5**). Indeed, 3-HT and BHT caused a significant decrease in IgE sensitization, which correlated with a trend toward diminished change in body temperature and mMCP-1 levels. Taken together, the results indicate that the effect of tBHQ on allergic sensitization and anaphylaxis is selective and not observed with other similar antioxidants.

### Adoptive transfer of Nrf2-deficient CD4^+^ T cells results in attenuated sensitization and anaphylaxis in comparison to wild-type CD4^+^ T cells

**Figure 6:**
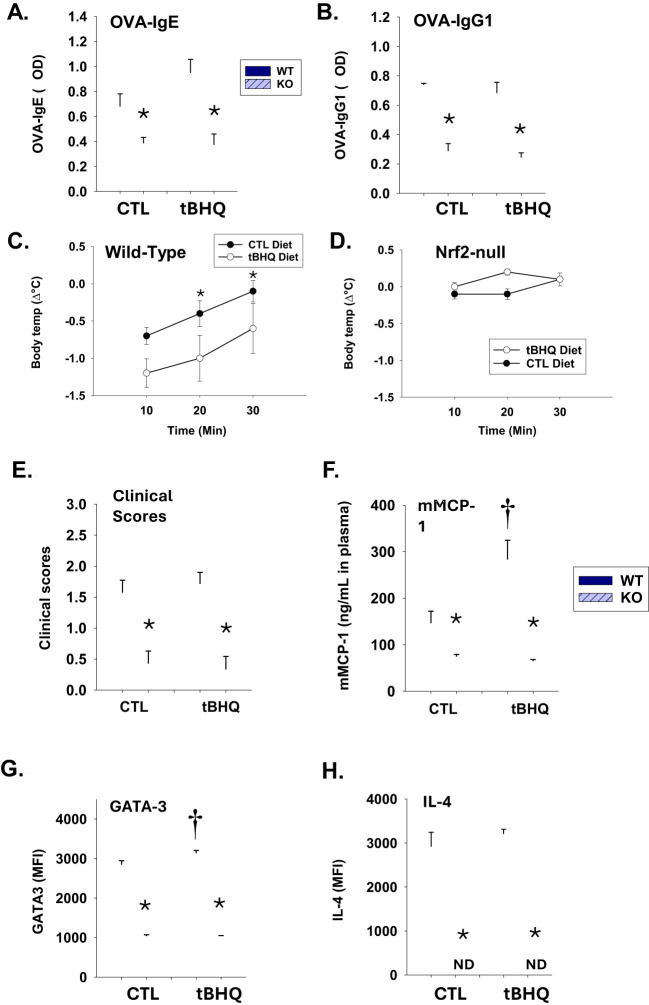
Sensitization and anaphylaxis to OVA is markedly diminished in SCID mice receiving Nrf2-deficient CD4^+^ T cells by adoptive transfer. SCID mice received wild-type or Nrf2-deficient CD4^+^ T cells via adoptive transfer (all animals received wild-type B cells) prior to OVA sensitization. Plasma levels of **(A)**: OVA-IgE (at 1/40 dilution) or **(B)**: OVA-IgG1 (at 1/2000 dilution) from individual animals were quantified by ELISA. *p < 0.05 as determined by two-way ANOVA with Bonferroni’s multiple comparisons test for *post hoc* analysis (n=7/WT on the control diet group, n=6/KO on the control diet group, n=7/WT on the diet with tBHQ group, n=7/KO on the diet with tBHQ). Data are presented as mean ± SE. **(C-H)** SCID mice receiving wild-type or Nrf2-deficient CD4^+^ T cells via adoptive transfer were challenged orally to OVA one week after the final sensitization. Rectal temperatures recorded before and at every 10 min after oral challenge in mice receiving **(C)** WT or **(D)** Nrf2-deficient CD4^+^ T cells. **(E)** Clinical scores of mice orally challenged with OVA (10 mg/mouse). **(F)** mMCP-1 plasma levels 1 h after oral challenge as determined by ELISA. The expression levels of **(G)** GATA3 and **(H)** IL-4 in CD4^+^ T cells as determined by mean fluorescence intensity by flow cytometry. Data are presented as mean ± SE. *p < 0.05 as determined by two-way ANOVA followed by Holm-Sidak or Bonferroni’s *post hoc* test (n=6-7). ND, Not detected. Statistical analysis for clinical scores between multiple groups was performed using a Kruskal-Wallis test with Dunn’s *post hoc* test.

Our previously published studies suggest that tBHQ promotes Th2 polarization through activation of the stress-activated transcription factor, Nrf2 (23). To investigate the role of Nrf2 in the effect of tBHQ on OVA-induced anaphylaxis, we implemented adoptive transfers of CD4^+^ T cells from wild-type or Nrf2-null mice and B cells from wild-type mice into SCID mice. The expansion of T cells in SCID recipients is no different between WT and Nrf2 KO (**Supplementary Figure 1**). The study revealed marked genotype differences. Of importance, recipients of Nrf2-deficient CD4^+^ T cells had markedly decreased plasma levels of OVA-IgE and OVA-IgG1 (**Figures 6A, B**). Strikingly, mice receiving Nrf2-deficient T cells did not become anaphylactic following OVA challenge (i.e., there was no drop in body temperature and no increase in clinical scores) (**Figures 6C–E**). Likewise, tBHQ caused an increase in OVA-IgE plasma levels and a greater drop in body temperature in mice receiving wild-type, but not Nrf2-deficient, CD4^+^ T cells, suggesting these effects are dependent on Nrf2. There were also notable genotype differences in Th2 cells and mast cells in which mice adoptively transferred with CD4^+^ T cells from Nrf2-null mice showed greatly attenuated IL-4 and GATA3 expression and decreased mMCP-1 release following OVA challenge (**Figures 6F–H**). Overall, these data suggest a critical role for Nrf2 during food allergen-induced sensitization and anaphylaxis.

### Adoptive transfer of Nrf2-deficient CD4^+^ T cells results in downregulated Th2-associated gene expression in comparison to wild-type CD4^+^ T cells

**Figure 7:**
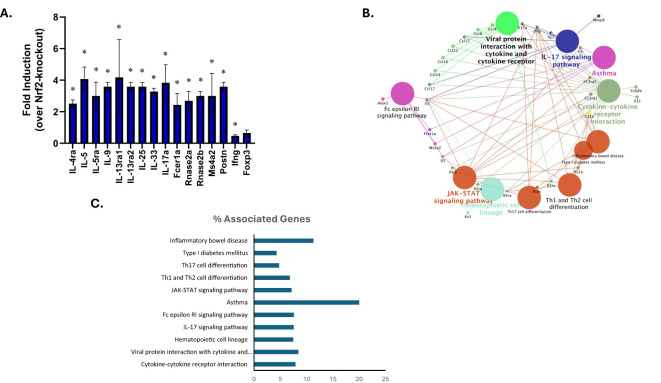
Intrinsic role for Nrf2 in modulating food allergy response as determined by effects on gene expression. SCID mice received wild-type (n=3) or Nrf2-deficient CD4^+^ T cells (n=3) via adoptive transfer (all animals received wild-type B cells) prior to sensitization and oral challenge to OVA. Wild-type and Nrf2-null splenocytes were analyzed to identify DEGs by using RT^2^ Profiler PCR Arrays Test. **(A)** Gene expression of representative DEGs were presented as fold induction as compared to Nrf2-null group. DEGs were determined by setting the threshold for fold change > 2 and P-values < 0.05. Data are presented as mean ± SE. *Student’s t test: p < 0.05 as compared to the Nrf2-null group. **(B)** Grouping of networks based on functionally enriched GO terms and pathways by using ClueGO, Cytoscape software. Pathway groups were generated based on Kappa Score = 0.5. **(C)** Percentage of genes associated with various pathways relative to the total number of associated genes as determined by KEGG analysis.

Wild-type and Nrf2-null splenocytes were analyzed to identify DEGs by using RT^2^ Profiler PCR Arrays Test. Compared with Nrf2-null group, DEGs were identified by setting the threshold for fold change > 2 and P-values < 0.05. Among the DEGs, expression of genes associated with Th2, mast cell, IgE receptor, and eosinophils were upregulated in the WT group, while the Th1-associated gene IFNγ and the Treg-associated genes Foxp3 was downregulated (**Figure 7A**, **Table 2**). To estimate the functions of identified DEGs, Cytoscape pathway and KEGG enrichment analysis was conducted, which revealed enrichment in pathways associated with asthma, inflammatory bowel disease, Th1 and Th2 differentiation, and Fc epsilon RI-mediated signaling (**Figures 7B, C**).

**Table 2:** The relative expression of genes in the wild-type group compared to Nrf2-null group.

	Fold Change^a^	T-TEST^b^
Symbol	Test Group /Control Group	p value
Adam33	**4.77**	**0.039143**
Adrb2	**2.90**	**0.000005**
Alox5	**2.48**	**0.000401**
Areg	1.61	0.148436
Arg1	1.75	0.063690
Bcl6	1.06	0.890472
Ccl11	1.95	0.211615
Ccl12	**3.58**	**0.000377**
Ccl17	2.25	0.060522
Ccl22	**3.70**	**0.001033**
Ccl24	**3.58**	**0.000400**
Ccl26	**2.61**	**0.003507**
Ccl4	0.92	0.846319
Ccl5	1.01	0.874036
Ccr3	1.98	0.183005
Ccr4	2.75	0.087689
Ccr8	**4.16**	**0.002766**
Cd40lg	1.15	0.381258
Chil1	5.44	0.086571
Chia1	**4.02**	**0.037128**
Clca1	**3.58**	**0.000377**
Cma1	1.38	0.236983
Cpa3	**1.89**	**0.035098**
Crlf2	**1.46**	**0.014673**
Csf2	0.68	0.411071
Csf3r	1.03	0.750825
Cysltr1	1.75	0.239560
Rnase2a	**2.69**	**0.009363**
Rnase2b	**3.58**	**0.000377**
Epx	0.73	0.243123
Ets1	**2.06**	**0.002733**
Fcer1a	2.43	0.054117
Foxp3	0.65	0.265743
Gata3	0.99	0.864250
Ptgdr2	1.22	0.504324
Icos	**1.89**	**0.005163**
Ifng	**0.46**	**0.003429**
Ifngr2	1.19	0.269668
Il10	**1.78**	**0.016682**
Il12a	**3.24**	**0.006743**
Il12b	**3.32**	**0.000607**
Il13	1.48	0.326604
Il13ra1	**4.18**	**0.013743**
Il13ra2	**3.58**	**0.000377**
Il17a	**3.83**	**0.040171**
Il17rb	1.81	0.239063
Il18	1.92	0.108628
Il1rl1	**1.92**	**0.022600**
Il21	1.25	0.286159
Il25	**3.58**	**0.000377**
Il2ra	**2.46**	**0.000009**
Il3	**3.58**	**0.000377**
Il33	**3.28**	**0.000493**
Il3ra	**2.79**	**0.004935**
Il4	1.74	0.111179
Il4ra	**2.51**	**0.001325**
Il5	**4.07**	**0.028192**
Il5ra	**3.49**	**0.014158**
Il9	**3.58**	**0.000377**
Itga4	**1.51**	**0.041910**
Kit	1.69	0.086469
Kitl	4.74	0.074055
Ltb4r1	**3.96**	**0.025986**
Maf	1.32	0.057786
Mmp9	2.95	0.052640
Mrc1	**2.88**	**0.026266**
Ms4a2	**3.00**	**0.028029**
Muc5ac	1.32	0.675084
Pdcd1	1.14	0.617865
Pmch	1.00	0.697150
Postn	**3.58**	**0.000377**
Pparg	2.25	0.250807
Prg2	1.17	0.244099
Retnlg	**4.40**	**0.001370**
Rorc	**2.53**	**0.001502**
Satb1	**1.78**	**0.015102**
Stat5a	1.17	0.201725
Stat6	**1.43**	**0.032183**
Tbx21	0.84	0.620042
Tgfb1	**1.62**	**0.048873**
Tnfrsf4	1.35	0.182266
Tnfsf4	2.63	0.078762
Tpsb2	1.86	0.214563
Tslp	0.90	0.947645
Actb	1.26	0.129747
B2m	0.43	0.054152
Gapdh	1.15	0.077181
Gusb	1.49	0.198705
Hsp90ab1	1.07	0.582211

^a^Fold-Change (2^(- Delta Delta Ct)) is the normalized gene expression (2^(- Delta Ct)) in the wild-type sample divided by the normalized gene expression (2^(- Delta Ct)) in the Nrf2-null Sample.

^b^The p values are calculated based on a Student’s t-test of the replicate 2^(- Delta Ct) values for each gene in the wild-type group and Nrf2-null groups. (n=3/each group).

Fold changes with a p value less than 0.05 are shown in bold font.

## Discussion

The current studies are the first to demonstrate that the stress-activated transcription factor, Nrf2, plays a critical role during sensitization and anaphylaxis in response to food allergen. Our studies are also the first to show that the common food preservative and Nrf2 activator, tBHQ, at concentrations relevant to human exposure, greatly exacerbates allergic sensitization as well as anaphylaxis to oral antigen. Specifically, our data provides direct evidence that tBHQ promotes OVA sensitization, elicits more allergenicity in mice, and exacerbates severe anaphylactic response to oral challenge. The presence of tBHQ in the diet was associated with an elevated type 2 T cell response with subsequent downstream effects, such as increased IgE production and mast cell degranulation. In addition, adoptive transfer experiments indicate that Nrf2 expression in CD4^+^ T cells is critical for the T cell response to food allergen.

Possible correlations between certain food additives and an increase in allergies have been proposed previously (32, 33). However, causation and mechanism have not been fully established. Here, our study on the effect of a phenolic antioxidant preservative tBHQ in OVA-elicited food allergy is the first to demonstrate that the food additive tBHQ promotes sensitization and exacerbates anaphylaxis in OVA-sensitized and -challenged mice and these effects likely occur through activation of the stress-activated transcription factor, Nrf2. In our model, the concentration of tBHQ was 0.0014%, which is the normal concentration of tBHQ in rodent feed. This concentration is less or equivalent to that found in many human foods (34, 35). The control diet used in the study was identical to the treatment diet except that the tBHQ was removed from the chow.

Numerous studies have established that tBHQ potently activates the stress-activated transcription factor, Nrf2, which drives expression of a battery of antioxidant and detoxification genes (36, 37). Accordingly, considerable research on tBHQ and Nrf2 has focused on the cytoprotective effects against acute toxicity and oxidative insult. However, a growing number of studies recognize a role for Nrf2 in modulating immunity. Our group was the first to demonstrate that activation of Nrf2 by tBHQ promotes Th2 differentiation and inhibits Th1 differentiation (23). Consistent with this, the present study shows an increased percentage of Th2 cells and Th2 cytokines in splenocytes from mice on the tBHQ diet in an ex vivo recall response to OVA.

The results from the PCR array suggest a significant role for Nrf2 in modulating type 2 immune response, even in the absence of exogenous Nrf2 activators. Specifically, expression of Th2 cytokines and their receptors were largely upregulated in the WT recipients compared to Nrf2-null group. Likewise, chemokines associated with type 2 response, such CCL24 (Eotaxin-2, CCL-26 (Eotaxin-3) and CCL-12 (MCP-5), were also upregulated in WT compared to Nrf2-null recipients. Unexpectedly, we also increased expression of genes associated with Th17 response, including RORγt and IL-17a. While a role for mixed Th2/Th17 responses has long been appreciated in asthma, there have been far fewer studies in food allergy and the results have been mixed, leaving the role of Th17 response in food allergy an open question at this time (38–40). Although IFNγ expression was significantly decreased in WT recipients compared to the Nrf2-null group, the expression of IL-12a and IL-12b was unexpectedly greater. However, it has been previously reported that IL-4 induces IL-12 expression by splenic and intestinal DCs (41). Antibody neutralization of IL-12 increased peanut-induced allergic sensitization and exacerbated anaphylaxis in response to crude peanut extract, suggesting a tolerogenic role for IL-12 (41). Collectively, the data suggest that Nrf2 promotes expression of genes associated with a mixed Th2/Th17 response in OVA-induced food allergy, however whether the Th17 response contributes to the pathology in this model is not currently clear.

The role of Nrf2 in type 2 immune responses appears to be complex and context-specific. In contrast to our current findings in an animal model of food allergy, other groups have reported that asthma is worsened in Nrf2-null mice. Specifically, whole body Nrf2-knockout mice showed increased inflammation, worsened airway hyperresponsiveness and elevated Th2 cytokine expression in OVA-induced asthma, which was attributed to the role of Nrf2 in airway epithelial cells in this model (42). In addition, clinical data suggests a correlation between low Nrf2 expression and increased severity in asthma patients (43). Conversely, an enhancer profiling study comparing humans with and without asthma revealed that Nrf2 is a strong positive determinant of Th2 differentiation (44). Interestingly, Brown et al. showed that the Nrf2 activator, sulforaphane, was protective in 60% of patients with moderate asthma, while exacerbating symptoms in 20% of patients (45). Taken together, the data point to a complicated role for Nrf2 in type 2 immune response in lung. Type 2 immune responses outside the lung differ markedly from those inside the lung with respect to mechanisms and cell types involved. There is significantly less data on the role of Nrf2 in type 2 immune responses outside the lung. However, Nrf2 promotes Th2 cytokine expression and infiltration of type 2-associated immune cells in an animal model of atopic dermatitis, which was diminished in Nrf2-null mice (46).

Recently, Wei et al. reported that Nrf2 activation by tBHQ may shift macrophages from classically activated macrophages (M1) to alternatively activated macrophages (M2) polarization by inhibiting NF-κB and promoting PPARγ expression in acute respiratory distress syndrome (47). M1 and M2 macrophages can promote Th1 and Th2 responses, respectively. This finding suggests that it is important to test how tBHQ regulates other types of immune cells through activation of Nrf2 such as dendritic cells, macrophages and other innate immune cells that can also impact T cell differentiation and allergy.

tBHQ activates Nrf2 through interaction with thiol groups on the Nrf2 negative regulator Keap1 which stabilizes the Nrf2 molecule (48). In addition to tBHQ, there are numerous other chemicals that can activate Nrf2 through a similar mechanism and thus Nrf2 is often considered a xenobiotic sensor. However, Nrf2 can also be regulated at the transcriptional and post-translational level (49, 50). The promoter region of Nfe2l2 (the gene which encodes Nrf2) contains xenobiotic response element-like (XRE) sequences, which are the binding sites of the aryl hydrocarbon receptor (AHR) (51). AHR is a basic helix-loop-helix transcription factor regulating xenobiotic metabolism and works in close concert with the transcription factor Nrf2. Published studies indicate that transcription of Nrf2 is directly modulated by AHR activation and conversely, Nrf2 regulates expression of AHR and subsequently modulates several downstream events of the AHR signaling cascade (51, 52). Thus, these two xenobiotic sensors regulate one another and both influence T cell polarization (23, 52–55). Of note, not all Nrf2 activators are expected to cause identical biological effects. There are multiple molecular mechanisms of Nrf2 activation, including differences in strength of interaction, numerous activator binding sites, different effects on Nrf2/Keap1 interaction, and other modes of transcriptional and post-translational regulation (49).

For these studies we chose to use skin exposure to induce allergic sensitization. There are many animal models of food allergy which employ various strategies to break oral tolerance, including use of genetically susceptible models (IL4raF709), strong adjuvants (cholera toxin or SEB, for example) and systemic i.p. sensitization (56, 57). Increasingly, investigators are using skin sensitization to bypass oral tolerance, which is the strategy used in the present studies. Each of these models has strengths and limitations. Genetic models and use of strong adjuvants allows for oral sensitization; however the downside is that most cases of food allergy in humans are not linked to cholera toxin exposure or defects in the IL-4 receptor. Skin sensitization has become popular because there is a correlation between atopic dermatitis and food allergy in humans (58). Furthermore, there is evidence to suggest that some infants and toddlers undergo an initial reaction upon first ingestion of a particular food, which indicates that sensitization likely did not occur via the oral route (59). Our data indicate that tBHQ enhances allergic sensitization via skin exposure, however it is not clear whether other routes of exposure would be similarly affected.

There are many different variations of skin sensitization used in animal models of food allergy, including presence or absence of adjuvant and intact skin vs. loss of barrier integrity via mechanical disruption or genetic mutation. Though the mechanism of how skin exposure to allergen induces sensitization is not fully characterized and may even be allergen-specific, recent studies suggest that mechanical injury could promote skin inflammation and induce Thymic stromal lymphopoietin (TSLP) and Th2 cytokines. In addition, published studies show that mechanical injury can polarize skin dendritic cells to elicit a Th2 response in certain food allergy models (60–62). Murine studies and clinical research indicate that both genetic mutation of proteins which maintain the integrity of the skin barrier (such as filaggrin) and the involvement of skin inflammation in the form of atopic dermatitis makes impaired skin barrier an important route for allergic sensitization (63–66). For the present studies, we used an adjuvant-free model of food allergy by sensitizing intact skin to antigen. Previous studies demonstrate that sensitization of intact skin to allergenic food proteins such as hazelnut, cashew nut, sesame, shellfish and wheat and ovalbumin results in clinical sensitization for oral anaphylaxis, which is consistent with what we observed with in our studies (26, 67–69). While atopic dermatitis can cause loss of barrier function, it is not clear whether the initial sensitization leading to dermatitis occurs in damaged or intact skin and whether this varies among individual patients. Thus, all of these models are useful and can be used in complementary ways to understand the association between skin exposure to antigen and the development of food allergy in humans.

Collectively, in this work, we provide the first evidence that Nrf2 mediates a type 2 T cell response in food allergy. Taken together with our previous study showing that activation of Nrf2 by tBHQ promotes Th2 differentiation *in vitro*, our research demonstrates that dietary tBHQ increases IgE sensitization and exacerbates anaphylaxis via a CD4^+^ T cell-Nrf2 dependent pathway.

## Data Availability

The original contributions presented in the study are included in the article/**Supplementary Material**. Further inquiries can be directed to the corresponding author.
