# Supplementary material for: The transcription factor Nrf2 links Th2-mediated experimental allergy to food preservatives
Source: Front Immunol. 2025 Mar 6;15:1476480. doi: 10.3389/fimmu.2024.1476480 (PMC11922944; doi:10.3389/fimmu.2024.1476480)
Supplement: Supplementary file 1 [file Presentation1.pptx]

## Slide 1
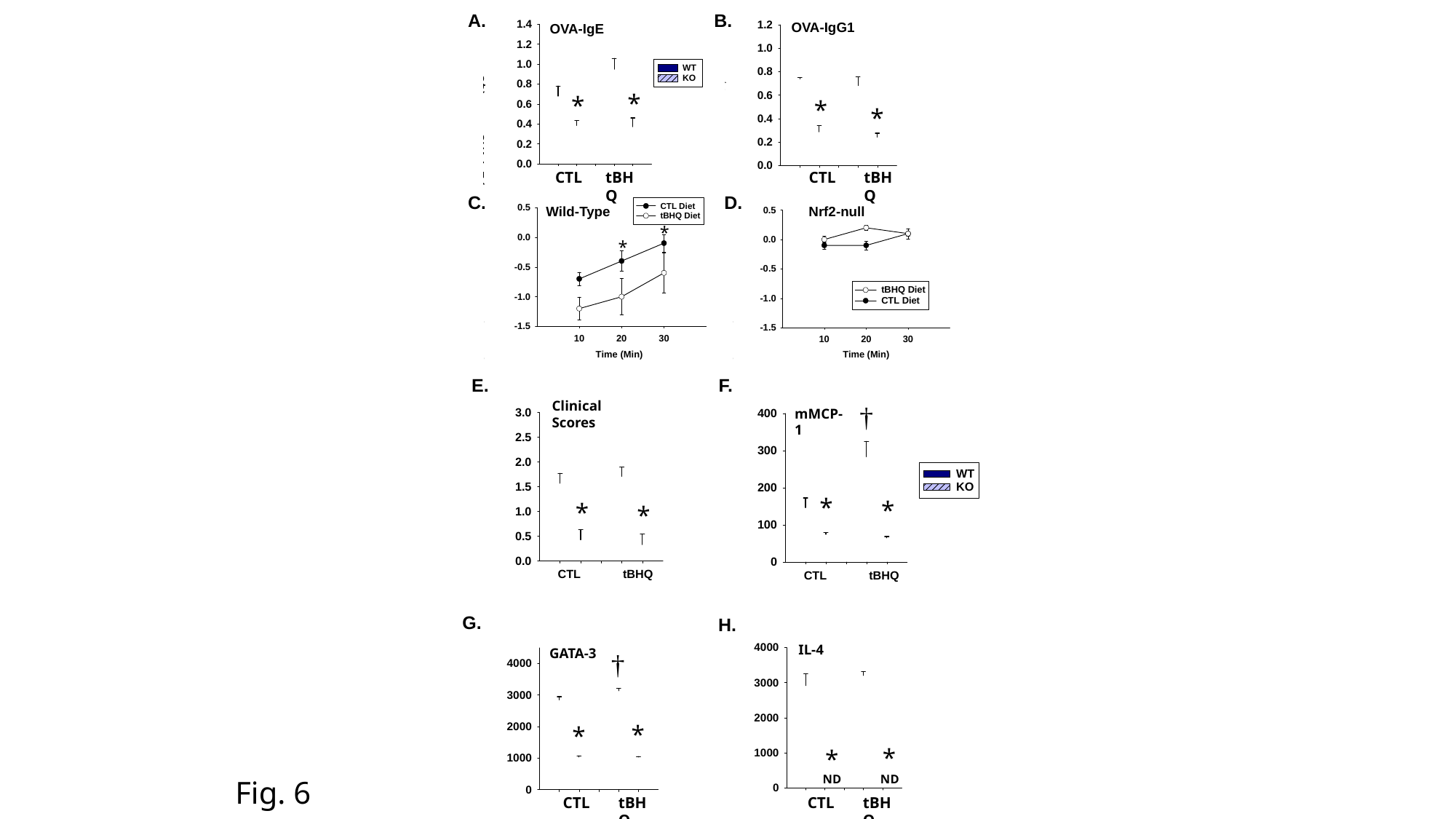

A.
B.
OVA-IgG1
OVA-IgE
*
*
*
*
CTL
tBHQ
CTL
tBHQ
C.
D.
Wild-Type
*
*
Nrf2-null
F.
E.
Clinical Scores
†
mMCP-1
*
*
*
*
G.
H.
IL-4
GATA-3
†
*
*
*
*
ND
ND
Fig. 6
CTL
tBHQ
CTL
tBHQ
